# Beyond the Moderating Role of Neuroticism on Evaluative Conditioning: Threat Appraisal

**DOI:** 10.1111/jopy.13017

**Published:** 2025-02-20

**Authors:** Darian Faur, Florin Alin Sava

**Affiliations:** ^1^ Department of Psychology West University of Timisoara Timisoara Romania

**Keywords:** evaluative conditioning, looming cognitive style, neuroticism, threat appraisal

## Abstract

**Objective:**

Reality perception is often altered by general dispositional factors that are associated with emotional vulnerability, both inherited and acquired, that emerge in a specific learning context. The current study will examine whether neuroticism and looming cognitive style, factors that account for emotional vulnerability, interact in a manner that influences the evaluative conditioning effect (the magnitude of valence change in a conditioned stimulus due to pairing with an unconditioned stimulus).

**Method:**

To achieve this, we will implement an evaluative conditioning procedure that pairs positive stimuli, nonthreatening negative stimuli, and threatening negative stimuli with neutral stimuli. Participants will also provide measures of valence and threat characteristics for the unconditioned stimuli, along with assessments of neuroticism and looming cognitive style.

**Results:**

We expect that the evaluative conditioning effect will be mediated by threat and valence evaluations of unconditioned stimuli. We also expect that neuroticism will moderate the valence and threat values of unconditioned stimuli, whereas cognitive looming will moderate the magnitude of valence transfer from unconditioned stimuli to conditioned stimuli.

**Conclusions:**

We presume that neuroticism will explain the reactivity to valenced and threatening stimuli, while looming cognitive style will account for the negative bias in the conditioned stimuli evaluation.

## 1 Introduction

A specific type of evaluation that helps one learn and predict the environment is evaluative conditioning (Hütter et al. 2014). It has been conceptualized as a change in the valence of a specific stimulus—a conditioned stimulus (CS)—due to its pairing with a positive or negative stimulus—an unconditioned stimulus (US; De Houwer 2007).

Due to the robustness of the evaluative conditioning effect (see Hofmann et al. 2010), it has been extensively used in research involving social and cognitive psychology (Moran et al. 2023). One of the theoretical perspectives on the evaluative conditioning effect is the propositional model (De Houwer 2018), which suggests that the formation of propositional knowledge in memory is mainly responsible for mediating the effect of the US valence on the CS evaluation. According to this perspective, individuals form propositions not only about the relation between the US and the CS (e.g., “the two images are displayed together”), but also about their attributes (e.g., “this image seems negative”). Recent studies that relied on this approach found that the US valence evaluation is a significant mediator of the evaluative conditioning effect (Ingendahl and Vogel 2023; Casini et al. 2023). That is, the deviations from the normed US valence depicted through the US evaluations seem to explain the evaluative conditioning effect.

Moreover, these mediative evaluations and the evaluative conditioning effect itself might vary between individuals due to their interpersonal differences. In this context, overarching factors such as personality traits may act as potential moderators of the effect. Among these traits, neuroticism stands out due to its direct connection to emotional reactivity (John and Srivastava 1999). With roots that can be found within the boundaries of classical psychoanalytical theory, neuroticism, in its modern understanding, was introduced by Eysenck with a focus on quick emotional arousal when stimulated and slow inhibition afterward (Ormel et al. 2013). Among the five domains that compose the Five‐factor model of personality or the Big Five, neuroticism is considered to be the personality trait that correlates the most with very intense emotional responses found in psychopathology (Kotov et al. 2010).

Previous studies considered neuroticism as a moderator for the evaluative conditioning effect. For example, Vogel et al. (2019) found that highly neurotic individuals tend to have a stronger predisposition for emotional learning (thus moderating the evaluative conditioning effect). This finding was later replicated by Casini et al. (2023), who explained the stronger emotional learning in neurotic individuals by the reaction (or evaluation) to the USs. However, when other traits are considered, this moderation effect becomes smaller, and only when the US evaluations are controlled does this effect emerge again (Ingendahl and Vogel 2023). Likewise, when ambiguity is addressed and inserted in the experimental paradigm, it reverts the approach to neuroticism as an emotional learning enhancer to a disposition that induces negative bias in learning (Bunghez, De Houwer, et al. 2024; Bunghez, Rusu, et al. 2024). Table 1 presents a summary of the findings regarding the relationship between neuroticism and the evaluative conditioning effect.

**TABLE 1:** Summary of the findings regarding interindividual differences in the evaluative conditioning effect.

Study	Neuroticism measure	Findings
Vogel et al. (2019)	German version of the Big Five Inventory	Stronger evaluative conditioning effects among neurotic individuals (mainly explained by the anxiety facet); the moderation effect, although significant, was small
Casini et al. (2023)	BFI‐2; HEXACO‐PI; HEXACO‐60; IPIP‐NEO‐120	Neuroticism strengthens not only the effects of negative USs but also of positive USs on the evaluative conditioning effect
Ingendahl and Vogel (2023)	BFI‐2	Neuroticism marginally moderates the evaluative conditioning effect and becomes stronger when the US evaluations are controlled
Bunghez, Rusu, et al. (2024)	NEO Personality Inventory; IPIP‐NEO‐120	Neuroticism moderates the evaluative conditioning effect in ambiguous scenarios, but not in unambiguously positive or negative situations
Bunghez, De Houwer, et al. (2024)	BFI‐2	Individuals with high levels of neuroticism tend to change the ratings of conditioned stimuli from positive to negative scenarios more significantly than from negative to positive scenarios in instruction‐based counterconditioning

Given the heterogeneity of the results regarding the role of neuroticism in the evaluative conditioning effect (i.e., does neuroticism function as a bias to negative evaluation of stimuli or as an emotional learning enhancer that leads to extreme positive and negative evaluations?), it might be useful to consider, in addition to the already existing valence appraisal (like or dislike) of the CSs (the traditional evaluative conditioning paradigm), other attributes that individuals might evaluate and also other subsequent factors that could moderate this evaluation.

### 1.1 Threat Evaluation

When confronted with an unconditioned stimulus, a person will most likely assess not only the valence of that stimulus (positive vs. negative) but will also infer other propositions about that stimulus (e.g., is it threatening or not). Threat evaluation is already a concept heavily relied upon in classical fear conditioning (see Duits et al. 2015), but it is not commonly used in evaluative conditioning procedures that only take into account the valence of the USs. The threat value of a stimulus is highly predictive of a subsequent fearful response, especially in a condition where the individual has already acquired the fearful response (Kreutzmann et al. 2021). Therefore, the threat evaluation of a stimulus could lead to a subsequent negative evaluation of another associated stimulus.

To the best of our knowledge, no prior studies examined the role of threat appraisal in valence evaluation (evaluative conditioning). However, previous authors have pointed out the fact that fear conditioning and evaluative conditioning are not independent but related to each other—the evaluations of the CS valence after an evaluative conditioning procedure affected the acquisition of fear in a subsequent fear conditioning procedure (Lipp et al. 2020). Similarly, changes in valence appear to predict subsequent fear (Dour et al. 2016). If valence and threat evaluations interact with each other, it is then plausible to presume that the threat ratings of the USs will further explain, in addition to the valence ratings, variability in the CSs evaluation regarding valence.

### 1.2 Negative Bias in Neutral Stimuli Evaluation: Looming Cognitive Style

So far, we have argued that focusing only on the valence of the stimulus might not be enough to explain the valence transfer to the neutral stimulus. Therefore, we proposed threat evaluation as a second mediator to be considered. Should threat evaluation be predictive of a subsequent negative evaluation of a neutral stimulus, then a cognitive bias that would explain interpersonal differences in the CS evaluation due to US evaluation would be a looming cognitive style.

Looming maladaptive or cognitive style biases an individual to perceive a stimulus as a rapidly increasing threat that is developing across space and time (Riskind 1997; Riskind et al. 2014). Because of its nature as a bias toward negatively evaluating a stimulus, looming cognitive style was particularly associated with anxiety—due to the overestimation of the threat of a specific stimulus, an individual who exerts this type of biased judgment will be prone to experience anxiety symptoms (Yeo et al. 2020). The theoretical foundation of the looming cognitive style draws on the concept of *prospection* (Seligman et al. 2013), which states that humans are mentally predisposed to represent possible futures or scenarios ubiquitously.

Both looming cognitive style and neuroticism are important factors for describing emotional vulnerability; thus, they can explain interindividual differences concerning emotional learning. They had previously been employed in research regarding emotional vulnerability, and their relationship appeared to be positively correlated, with studies reporting low to moderate associations (Hong 2013; Zamani et al. 2022). While it has been argued that looming cognitive style has a weak contribution in predicting specific vulnerabilities (emotional responses), especially when other social‐cognitive biases are controlled (Hong 2013), it should nonetheless function as a moderator of the CS evaluations because it is conceptualized as a proximal cognitive vulnerability that directly influences anxious responses (or negative evaluations), while neuroticism, as a personality trait, is a general predisposition to emotional reactivity (Olino et al. 2024).

Not only does the looming cognitive style concretize the general emotional reactivity explained by neuroticism, but it is also directly related to threat evaluation: looming induces a biased threat processing that is mainly specific to anxiety syndromes (Yeo et al. 2020; Riskind 2024). Therefore, based on theory and previous findings, we presume that, in an experimental situation where participants are faced with visually depicted threatening stimuli (instead of a mere self‐reported measure of anxiety), the looming cognitive style would emerge as a significant moderator, influencing the intensity of the CSs evaluation.

### 1.3 The Current Study

**FIGURE 1:**
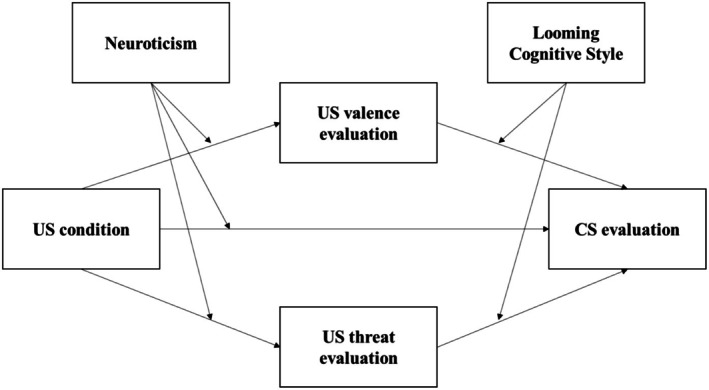
Conceptual diagram of the parallel moderated mediation model.

In the present study, we want to examine how factors that account for interpersonal differences (neuroticism and looming cognitive style) interact in a model that considers valence and threat evaluation as mediators for the evaluative conditioning effect. To better understand the complexity of this interaction, we will investigate the existing evidence in favor of a multilevel moderated mediation. The conceptual model can be visually inspected in Figure 1.

We anticipate that the evaluation of valence and threats associated with the US will mediate the impact of the assigned valence of the US on the evaluation of the CS. Additionally, we consider that neuroticism will account for the intensity of US evaluations—specifically, responses to valenced and threatening stimuli—while looming cognitive style will explain the negative bias observed in CS evaluations. On a practical level, we presume that, in line with findings from Casini et al. 2023, neuroticism acts as an enhancer of US individual evaluations (for valence and threat characteristics), while the looming cognitive style interacts with these evaluations to induce a negative bias in the CS evaluations. While neurotic individuals will react more strongly to valenced and threatening stimuli compared with low neurotic individuals, the presence of the looming cognitive style in individuals will cause their negativity bias in the CS evaluations.

Since individuals react differently to the environment, they tend to infer different characteristics from the specific learning context in which the stimuli occur. Because personality plays a significant role in these interindividual differences, characteristics such as positive or negative, threatening or nonthreatening, salient or nonsalient of one stimulus will be appraised differently especially if an emotional vulnerability‐related trait is present at a higher level. Thus, linking personality to other factors more proximal to specific symptoms might offer important insights into the emotional learning processes involved in psychopathology.

The importance of this approach relies on two aspects. On the one hand, this approach makes the evaluative conditioning effect suitable for the clinical context. It adds incremental value in understanding how the interaction between personality, social‐cognitive factors, and the learning experience interacts so that internalized psychopathology could emerge or be maintained. Since stimuli are not only evaluated based on their valence but also based on other attributes, threat appraisal may offer supplemental information concerning the evaluative conditioning effect in a certain context. Research on this topic has only focused on the valence of the stimuli, despite previous findings suggesting that our perception is more prone to a stronger and quicker reaction to stimuli that hold a threatening instance compared with negative stimuli that are not considered to be threatening (March et al. 2017). The insertion of threat appraisal comes into the picture to emphasize the role of valence transfer in the development of internalized disorders.

On the other hand, as previously argued, it considers neuroticism as a distal factor that influences one's ability to appraise a certain stimulus via a more specific, proximal social‐cognitive factor. This has strong theoretical foundations and is consistent with Barlow's triple vulnerability model of internalizing disorders (Barlow 2000), which argues that the foundation of internalized psychopathology relies on two generalized vulnerabilities (biological—neuroticism and psychological—a diminished sense of control) and one specific vulnerability based on learning experiences. In our case, looming cognitive style, the overarching social‐cognitive factor related to anxious responses, is supposed to explain CS evaluations above the emotional reactivity induced by neuroticism, the generalized vulnerability, to the specific learning instance, that is, the evaluative conditioning procedure. People scoring high in looming cognitive style tend to perceive threats as more imminent or more serious, therefore increasing the stimulus salience/relevance. Thus, people who score both high in neuroticism and looming cognitive style could not only react more intensively to USs valence but could also transfer this valence to a higher extent to the corresponding CSs. Regularly, this could be an adaptive process of learning faster to transfer valence from USs to CSs when USs stimuli are not only negatively valenced but are also perceived as more threatening. However, when a person tends to overreact to valence stimuli because of high levels of neuroticism, and also to perceive the threat as more serious because of high levels of looming cognitive style, the risk of false positive reactions is increased, and it is phenomenologically expressed as an orientation toward the negative aspects of life and a tendency to experience more often negative emotions.

### 1.4 Hypotheses

Regarding the first level of the moderated mediation model (the within‐person dimension), our first hypothesis (H1a) will be that there is an evaluative conditioning effect (the US condition levels on the CS evaluation). Then, we will address this effect in a mediation model with two parallel mediators: We presume that the evaluative conditioning effect is explained by the US valence evaluation (mediator 1, H1b) and by the US threat evaluation (mediator 2, H1c). To summarize our expectations for the within‐person level, we presume that the evaluative conditioning effect will be explained by the individual evaluations of valence and threat attributes of the USs.

At the second level of the model (the between‐person dimension), we expect this evaluative conditioning effect (the effect of the US condition on CS evaluation) to be enhanced by the interaction between the US condition and neuroticism (H2). Likewise, we expect that both the US valence evaluation and the US threat evaluation are moderated by neuroticism; that is, we expect that the interaction between the US condition and neuroticism will predict the US valence evaluation and the US threat evaluation (H3). Finally, we will examine whether the effect of US valence evaluation and US threat evaluation on the CS evaluation is moderated by the looming cognitive style (H4). To summarize our hypotheses for the between‐person level, we presume that neuroticism will increase reactivity to normatively valenced and threatening stimuli, while the looming cognitive style will negatively bias CS evaluations.

## 2 Pilot Study

For the purpose of the current pilot study, we chose a convenience sample of *N* = 100 participants that would assure differentiation between the positive, negative, nonthreatening, and negative‐threatening stimuli. The sample for the pilot study will be composed of participants who are at least 18 years of age and are recruited from the general population. The pilot study aims to carefully distinguish between negative nonthreatening stimuli and negative‐threatening stimuli. This classification will facilitate their inclusion in the upcoming experimental procedure. In light of the fact that we are not making any prior assumptions regarding the results, and since the conditioning paradigm will not be incorporated in this phase, we have chosen a more straightforward approach and opted not to conduct size estimations at this time.

To correctly assign the USs to each targeted category, we will conduct a pilot study similar to March et al. (2017). We will select 100 pictures from the 900‐picture Open Affective Standardized Image Set (OASIS; Kurdi et al. 2017) and present them in a random order. We will not include here pictures that are normatively judged as neutral (based on the OASIS ratings), and we will preselect three categories of USs: positive USs, low‐threat negative USs, and high‐threat negative USs. The participants will rate each of the presented images on three dimensions: positive valence, negative valence, and threatening valence on a 7‐point Likert scale (ranging from 1 = *not at all* to 7 = *extremely*).

After computing the means for the three types of valences for each image, we will assign the images into three categories, as follows: *positive USs* (negative and threat ratings lower than 3.00 and positive ratings greater than 5.00), *negative nonthreatening USs* (positive ratings less than 3.00, negative ratings greater than 5.00, and threat ratings less than 3.00), and *negative‐threatening USs* (positive ratings less than 3.00, negative ratings greater than 5.00 and threat ratings greater than 5.00).

The final step of the stimuli selection will consist of excluding the images that are not clearly visible on a 4.17 × 3.15 in. frame and selecting two final USs from each category (two for positive, two for negative nonthreatening, and two for negative threatening) that will be included in the experiment so that the negative USs will be similar in terms of valence, but they will maximize the difference in threatening rating.

## 3 Method

### 3.1 Participants and Design

#### 3.1.1 Sample Characteristics

Participants in this experiment will be subjects of at least 18 years of age, recruited via the Prolific platform.

#### 3.1.2 Sample Size Estimation and Exclusion Criteria

Participants who fail to rate any of the CSs (grayscale fractals) as neutral in the preconditioning phase and those who lack variability in their responses (for any measurement, including neuroticism and looming cognitive style) will be excluded from the experiment. These criteria have been previously employed in other evaluative conditioning effect experiments that address the moderating role of personality traits (see Vogel et al. 2019; Casini et al. 2023) for their role in assuring a nonbiased posttest evaluation.

In estimating our sample size, we conducted two analyses: one for the level‐one parallel mediation model and another that accounts for the level‐two analysis where we introduce moderators. For the first level, we employed the simulation approach proposed by Schoemann et al. (2017). This approach included one predictor (US condition), two parallel mediators (US valence evaluation and US threat evaluation), and one outcome (CS evaluation). With a sample size of *N* = 305, the model achieves a power of 82%, assuming the average correlations among the four variables (US condition, US valence evaluation, US threat evaluation, and EC effect) are *r* = 0.24. We based this average correlation coefficient on findings from Casini et al. (2023).

For the second level, we ran an a priori power analysis in GPower (Faul et al. 2007) using the mixed ANOVA design with two groups and three repeated measures (for a similar a priori power analysis, see Ingendahl and Vogel 2023). We approximated our design by considering neuroticism as a continuous between‐subject predictor and the US‐assigned valence as a within‐subject predictor. The a priori analysis revealed a minimum sample of *N* = 798 required to obtain 80% power for a small effect size of *f* = 0.11 (standard *α* = 0.05). We estimated the small effect size based on previous studies that tackled this issue and generally estimated for their samples and then reported based on the results a small moderation effect of neuroticism on the evaluative conditioning effect (see Table 1 for references; a mean was computed between estimated effect sizes reported by these authors).

Because the present study introduces additional variables and, subsequently, increases the measurement error, we will aim for at least *N* = 800 participants that will ensure enough power for the analyses.

#### 3.1.3 Design

The experimental procedure will include repeated measures (three‐level within‐person independent variable—US condition: positive vs. negative nonthreat vs. negative threat) mixed‐effects model with moderated mediation.

### 3.2 Materials

#### 3.2.1 Self‐Reported Measures

The demographic data we plan to collect include information about gender, age, residential area, and educational background.

Neuroticism will be measured with IPIP‐NEO‐120 (Johnson 2014). Using a 5‐point Likert scale ranging from 1 (strongly disagree) to 5 (strongly agree), the instrument has 24 items that capture six facets: Anxiety, Anger, Depression, Self‐consciousness, Immoderation, and Vulnerability.

The LMSQ‐R (Riskind et al. 2000) will be selected to measure the looming cognitive style. The instrument has six vignettes depicting scenarios of rapidly increasing anxiety. Each vignette is measured on a 5‐point Likert scale, capturing both social and physical components of the looming cognitive style.

#### 3.2.2 Evaluative Conditioning Procedure

For the CSs, we will initially use a larger pool consisting of 20 computer‐generated grayscale fractals that were previously used in similar evaluative conditioning procedures (e.g., Sava et al. 2020). In the preconditioning phase, the participants will rate all the CSs, and we will select the six CSs that receive the most neutral evaluations for each participant (i.e., closer to 0 on the employed scale).

From these six CSs, two of them will be paired with positive USs, two with negative nonthreatening USs, and the remaining with negative‐threatening USs. The USs will be images from the OASIS, assigned in each of the mentioned categories after the pilot study, and the materials will be presented on a computer screen via Inquisit 6 software. To more effectively convey the looming cognitive style through our stimuli, we decided to transform the static images of the USs into dynamic images featuring a slow zoom effect. This approach highlights the looming bias, which perceives the threat as not only approaching but also increasing in intensity.

### 3.3 Procedure

The first part of the procedure will involve providing demographic information (gender, age, residence area, and educational background) as well as neuroticism and looming cognitive style questionnaires. The study will be presented as a visual perception task.

The evaluative conditioning procedure will follow. Our evaluative conditioning paradigm is an adaptation of Vogel et al. (2019) that was, in a similar fashion, adapted from Hütter et al. (2012). It consists of three main phases, described as follows:

#### 3.3.1 CSs and USs Evaluation Phase

As mentioned, in the initial phase, each participant will evaluate 20 CSs, and only six will be selected for the conditioning procedure (the most neutrally rated stimuli). The employed rating scale will range from −7 (very unpleasant) to 7 (very pleasant). Therefore, the final six CSs will be the ones that are closer to zero. This will not only serve as a control measure for tailoring specific CSs to each participant, but also as an exclusion criterion: The participants who fail to rate at least six CSs as neutral (a score outside the −2 to 2 interval) will be deemed unsuitable for our study.

After the CS's initial rating, the participants will evaluate the US valence using the same scale previously employed in the CS evaluation phase. In addition, participants will evaluate their threat perception regarding the US.

#### 3.3.2 Conditioning Phase

After the evaluation phases, the conditioning task will begin. Each CS will be paired with one US in a one‐to‐one pairing strategy. The CSs will always be displayed on the left side of the screen. With respect to dimensions, the CSs frames will measure 3.15 × 3.15 in., while the USs frames will measure 4.17 × 3.15. To implement the zoom effect, we will gradually increase the size of the frames for both the USs and CSs throughout the entire presentation. The zoom will progress at a rate that ensures the frame size is magnified by at least 50% by the end of the presentation.

**FIGURE 2:**
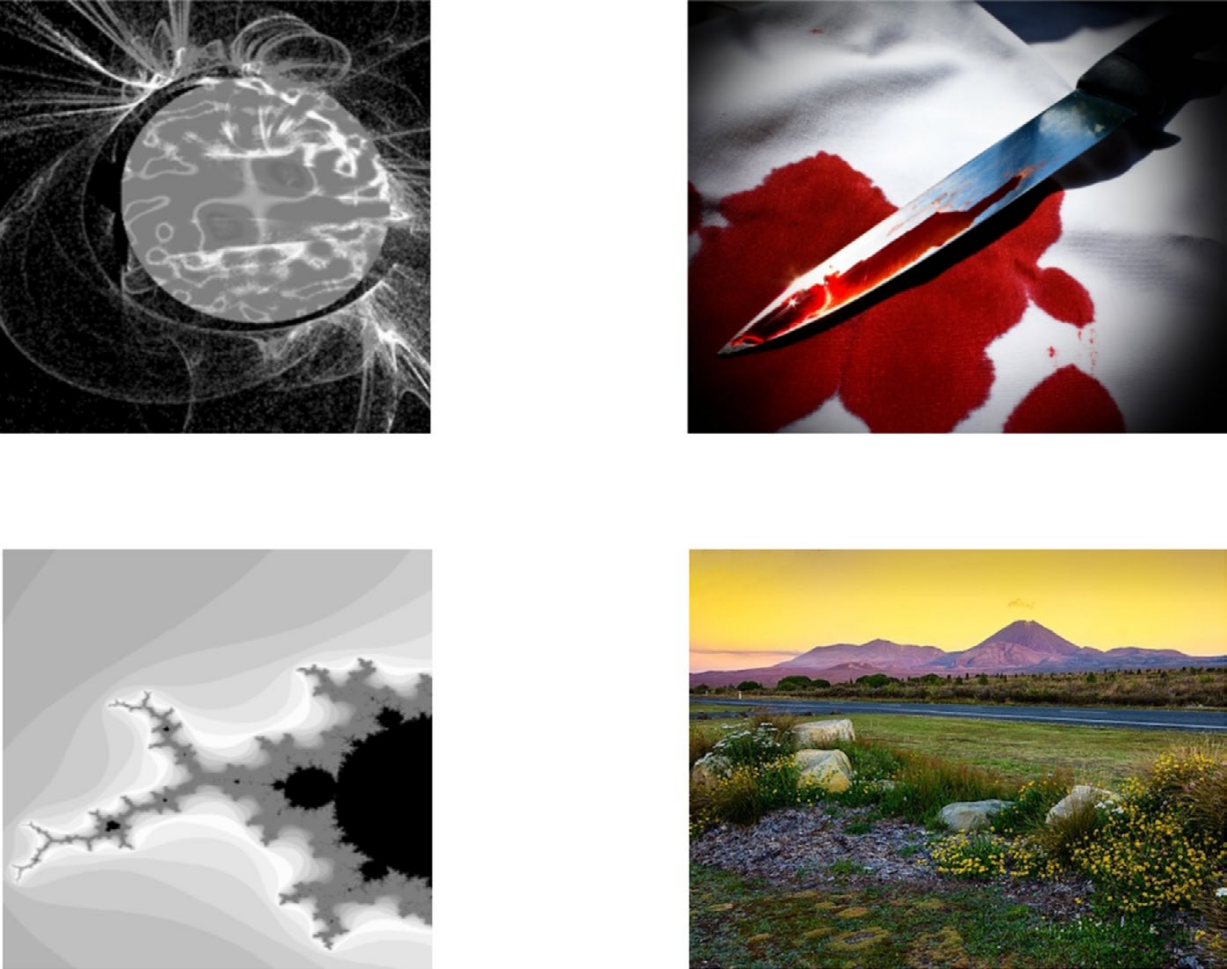
Visual representation of the evaluative conditioning procedure. The CSs are displayed on the left and are portrayed by the grayscale fractals. The USs are displayed on the right side. There will be a single CS–US pair presented each time.

The EC task will consist of 6 cycles, resulting in 36 trials. Each CS‐US pair will be presented on the screen for 4000 ms with an interstimulus interval of 1000 ms. Figure 2 illustrates the evaluative conditioning procedure using two CSs (the grayscale fractals), one negative US (according to the OASIS ratings), and one positive US (OASIS ratings).

#### 3.3.3 Postconditioning Phase

After the experimental procedure, the participants will rate once again the CSs using the same scale, ranging from −7 (very unpleasant) to 7 (very pleasant). A contingency awareness task will also be administered by asking participants to link the presented CSs with the USs they remember forming a pair in the previous phase. In order to reduce the chance of randomly assigning the pairings, the locations of the correct and incorrect USs will be randomized across the screen. A short debriefing regarding the main purpose of the experiment will be presented at the end.

### 3.4 Ethics and Open Science Policy

This study will be undertaken after receiving the approval of the institutional ethical committee. To assure transparency and openness for our research, we will make the database and the analysis files openly available on the Open Science Framework (OSF; https://osf.io/). Anonymizing the participants in the database will assure confidentiality.

### 3.5 Planned Analyses

#### 3.5.1 Preliminary Analyses

All analyses will be conducted in R (version 4.3.0). Regarding the reliability of the instruments, we will report internal consistency indices (both Cronbach's alpha and McDonald's Omega).

We will test the assumptions of regression and mediation models by checking for the linearity between the mediators and the outcomes, normality of the residuals (using QQ plots), and multicollinearity (variance inflation factor—VIF). We will also check the distribution of the outcome variable (CS evaluation) and the two mediators (US threat evaluations and US valence ratings) in order to check for normality. Bivariate correlations will also be explored in the preliminary analyses section so that we can get a sense of the relationships involved in the main model.

Prior to conducting our main data analyses, we will create dummy variables for the US condition. Specifically, the positive US level will serve as the reference category, while the other two levels—negative nonthreatening and negative threatening—will be dummy‐coded accordingly. This coding scheme allows for direct comparisons between each of the two negative conditions and the positive reference condition in subsequent analyses.

#### 3.5.2 Main Data Analysis and Hypothesis Testing

Because our model will involve a multilevel approach, the analyses will be conducted using the *lmerTest* package (Kuznetsova et al. 2017) and the *bruceR* package in R (Bao 2022).

At Level 1 (the within‐person dimension), we will test the mediation model where the outcome (CS evaluation) is predicted by the mediators (US valence evaluation and US threat evaluation) and by the US condition (dummy‐coded). Regarding the random‐effect structure, because we will have six repeated measures for every participant, we will specify subject‐level random intercepts, which will model the baseline differences in CS evaluation across participants (baseline level of CS evaluation).

At level 2 (the between‐person dimension), we will add the moderators in the following manner: neuroticism will interact with US condition to affect both mediators (US valence evaluation and US threat evaluation), which corresponds to the *a* path in the mediation model, and also to affect the CS evaluation (which corresponds to the *c'* path or the direct path in the mediation model). On the other hand, looming cognitive style interacts with US valence evaluation and US threat evaluation (the two mediators) to influence the CS evaluation (which would correspond to path *b* in the mediation model). Regarding the random effects of the second level, we will keep the random intercepts (baseline CS evaluation for each participant) and we will add random slopes for the predictor (US condition) and the two mediators.

Should the null results emerge from the analysis, we also plan to implement Bayesian analysis alongside our standard analyses by computing the Bayes factor, which quantifies the evidence for one hypothesis relative to another. For this purpose, we will use the Bayes Factor package in R (Morey et al. 2018).

This analysis could offer insights into the informativeness of the potential null effects. For example, the absence of a mediating role for US valence, combined with the presence of a mediating role for US threat in the evaluative conditioning (EC) effect, would suggest that humans can transfer valence from a US to a CS more quickly when the US is perceived as threatening—an adaptive feature. Conversely, if this effect is moderated by Neuroticism, such that individuals with high Neuroticism scores are less discerning and transfer valence to a lesser extent in threatening conditions compared to nonthreatening ones, this could offer valuable theoretical insights. Methodologically, null findings might indicate the need to adjust the standard approach for examining how individual differences moderate the EC effect. For instance, incorporating a mood activation task prior to the conditioning phase could help activate traits like neuroticism or a looming cognitive style.

## Author Contributions

**Darian Faur:** conceptualization, methodology, writing – original draft, writing – review and editing. **Florin Alin Sava:** conceptualization, supervision, methodology, writing – review and editing.

## Conflicts of Interest

The authors declare no conflicts of interest.
